# Adapting tissue-engineered in vitro CNS models for high-throughput study of neurodegeneration

**DOI:** 10.1177/2041731417697920

**Published:** 2017-03-15

**Authors:** Caitriona O’Rourke, Charlotte Lee-Reeves, Rosemary AL Drake, Grant WW Cameron, A Jane Loughlin, James B Phillips

**Affiliations:** 1 Department of Biomaterials and Tissue Engineering, UCL Eastman Dental Institute, University College London, London, UK; 2 Department of Life, Health and Chemical Sciences, The Open University, Milton Keynes, UK; 3 TAP Biosystems, Royston, UK

**Keywords:** Neurodegeneration, three-dimensional models, neurons, drug screening, induced pluripotent stem cells

## Abstract

Neurodegenerative conditions remain difficult to treat, with the continuing failure to see therapeutic research successfully advance to clinical trials. One of the obstacles that must be overcome is to develop enhanced models of disease. Tissue engineering techniques enable us to create organised artificial central nervous system tissue that has the potential to improve the drug development process. This study presents a replicable model of neurodegenerative pathology through the use of engineered neural tissue co-cultures that can incorporate cells from various sources and allow degeneration and protection of neurons to be observed easily and measured, following exposure to neurotoxic compounds – okadaic acid and 1-methyl-4-phenylpyridinium. Furthermore, the technology has been miniaturised through development of a mould with 6 mm length that recreates the advantageous features of engineered neural tissue co-cultures at a scale suitable for commercial research and development. Integration of human-derived induced pluripotent stem cells aids more accurate modelling of human diseases, creating new possibilities for engineered neural tissue co-cultures and their use in drug screening.

## Introduction

Neurodegenerative diseases remain a poorly understood and growing public health concern, mired by the consistent failure to see advancements in therapeutic research through to successful clinical trials.^1^ The principal risk factor for the development of neurodegenerative disease is ageing; thus, it is becoming increasingly important to advance neurodegenerative research as the older population continues to increase. The most common neurodegenerative condition worldwide is Alzheimer’s disease, which presents with cognitive, behavioural and emotional decline, and the second most common is Parkinson’s disease, which primarily affects global motor function.^2^

Fundamentally, these diseases bring about the loss of function and eventual death of neurons; however, the mechanisms by which this can occur are numerous and complex. Neurodegenerative diseases typically involve a series of systemic, cellular and molecular processes that can be difficult to isolate and examine individually. Despite distinct clinical presentations, many of the sub-cellular aspects are shared between different disease types, such as protein misfolding and aggregation,^3^ and this is also true for the destructive mechanisms that become a steadily progressive and self-sustaining cycle.^4^ The physical degeneration that occurs prior to the death of neuronal cells first involves the loss of the connections between them; the axons and dendrites (neurites) that help develop and maintain the vast and intricate communicative network of the brain. Seidel et al.^5^ define this process as occurring over four distinct sequential phases: axonal swelling, neurite retraction, cell swelling with neurite degeneration and cell death.

Aspects of neurodegenerative pathology can be replicated using a selective protein phosphatase 2A inhibitor, okadaic acid, which is commonly used to induce tau hyperphosphorylation in vitro.^5–7^ Okadaic acid is an environmental marine toxin released by shellfish that inhibits protein phosphatases that are involved in a number of vital cellular processes. Recently, its neurotoxic effects have been recognised with observations of degenerating neurons in vitro,^8,9^ and it was demonstrated to induce memory impairment through mitochondrial dysfunction and apoptosis in vivo.^9,10^ 1-Methyl-4-phenylpyridinium (MPP^+^), an oxidative neurotoxic metabolite of 1-methyl-4-phenyl-1,2,3,6-tetrahydropyridine (MPTP),^11–13^ is widely used in vivo and in vitro for disease modelling to induce symptoms of Parkinson’s disease in animal models. MPP^+^ has been found to specifically target dopaminergic neurons as it interferes with the oxidative phosphorylation within mitochondria thereby depleting ATP and causing apoptosis.

Three-dimensional (3D) central nervous system (CNS) cell culture models are a valuable research tool as they create a physiologically relevant environment for cells allowing them to behave as they would in vivo, capturing some of the complexity of the in vivo environment and allowing cellular changes to be monitored, analysed and quantified. They are able to recapitulate some of the mechanical cues provided by the extracellular matrix environment in vivo. In terms of neurodegeneration, 3D models can accurately simulate the physical degenerative processes of neurons and allow for degeneration to be closely assessed.^14–21^ Additionally, these models can employ glial cells in co-culture to provide a closer approximation of the in vivo environment.

Tissue engineering provides the opportunity for development of artificial nervous system tissue for use in regenerative medicine and for in vitro modelling,^17,22,23^ and there is increased interest in the development of advanced tissue models that organise mixtures of neurons and glia in co-culture. ‘EngNT’ (engineered neural tissue) incorporates self-aligned chains of glial cells within a collagen gel and has been used in nervous system regenerative medicine.^24–28^ We propose that EngNT has the potential to form the basis of a 3D CNS tissue model in which appropriate CNS cells can be incorporated. The alignment of neurites in EngNT facilitates quantification of neurite length and may provide a potentially useful platform for simulating neurodegeneration.

This study initially uses EngNT containing co-cultures of glial cells and either the neuronal cell line PC12 or primary rat dorsal root ganglia (DRG) neurons to create artificial nervous system tissue models. Additionally, the bioactive compound salvianolic acid B (SAB) was used as a treatment due to its previously reported neuroprotective antioxidant effects,^21^ to determine whether the model may be suitable for drug screening. Subsequently, we miniaturised the model system to a scale more suited to industrial purposes and demonstrate how cell types more relevant for studying human disease such as human induced pluripotent–derived stem cells can be incorporated effectively.

## Materials and methods

### Cell culture

Astrocytes from the rat C6 cell line (ATCC^®^CCL-107™) were maintained in culture medium (Ham’s F12; Gibco) supplemented with penicillin and streptomycin (100 U/mL and 100 mg/mL, respectively; Sigma) and 10% v/v foetal calf serum (Sigma) in standard cell culture flasks.

PC12 cells (rat neuronal cell line, 88022401; Sigma) were grown in suspension in culture medium (RPMI 1640; Sigma) supplemented with penicillin and streptomycin (100 U/mL and 100 mg/mL, respectively; Sigma), 2 mM l-glutamine, 10% v/v heat-inactivated horse serum and 5% v/v foetal calf serum (Sigma) in standard cell culture flasks.

Human induced pluripotent stem cell (iPSC)–derived neural progenitor cells (NPCs; ax0015; Axol) were cultured on laminin-coated flasks (20 µg/mL; Millipore) and expanded to passage 5 with neural expansion XF-media (ax0030-50; Axol) supplemented with epidermal growth factor (EGF) and fibroblast growth factor (FGF; 20 ng/mL; Millipore), with a media change every 2 days.

Primary DRG neurons were dissected from adult rat spines. Nerve roots were stripped and DRGs were incubated in 0.125% collagenase (Sigma) for 2 h at 37°C. Tissue was dissociated by trituration and washed twice by centrifugation with 10 mL of culture medium (Dulbecco’s Modified Eagle Medium (DMEM); Sigma) also supplemented with penicillin and streptomycin and foetal calf serum for 5 min at 250*g* to remove any remaining collagenase.

### Fabrication of EngNT co-cultures

All gels were prepared using 80% v/v Type I rat tail collagen (2 mg/mL in 0.6% acetic acid; First Link, UK) mixed with 10% v/v 10× minimum essential medium (Sigma), and the mixture was neutralised using 5.8% v/v neutralising solution (Lonza Bioscience) before addition to 4.2% v/v cell suspension (3 × 10^6^ C6 cells/mL of gel). The collagen mixture was added to various moulds as given below:

1. Rectangular mould (16 mm long) integrated with tethering mesh at opposite ends as described previously,^20,29^ which required 1 mL of collagen mixture and was termed ‘1-mL gel’.
2. A custom-designed scaled down (6 mm long) version of the mould described above that requires 50 µL of collagen mixture, 20 times less material and fewer cells and was termed ‘50-µL gel’

All gels were allowed to set at 37°C for 15 min. Cellular gels were immersed in culture medium and incubated at 37°C in a humidified incubator with 5% CO_2_/95% air for up to 24 h, during which time the C6 cells become aligned.^30^ Using RAFT absorbers (Lonza Bioscience), the aligned gels were stabilised for 15 min (1-mL gel) or 3 min (50-µL gel). Stabilisation is a process whereby a biocompatible material is placed upon a gel and slowly absorbs interstitial fluid to generate a dense robust hydrogel with a 50-fold increase in cell and collagen density. PC12 cells, iPSC or DRG neurons (75,000 per gel) were cultured on the surface of the stabilised collagen gel and maintained in culture for 3 days to allow for neurite growth before treatments were applied. PC12 cells were incubated with nerve growth factor (100 ng/mL; Sigma) while iPSCs were maintained in neural differentiation XF-media (ax0034-125; Axol) to promote neuronal differentiation.

### Neurotoxicity

After 3 days, culture medium was removed and replaced with either 3 mL fresh Ham’s F12 media (control) or 3 mL Ham’s F12 media with added neurotoxin for 24 h, before fixing in 4% paraformaldehyde (PFA) at 4°C. Okadaic acid (Calbiochem; gift from Dr Brian Pearce, UCL) stock solutions were prepared in dimethyl sulfoxide (DMSO) and final concentrations of 5, 25 and 50 nM^5^ were diluted in cell culture medium. MPP^+^ (Sigma) was prepared in cell culture medium at concentrations of 1, 10, 30, 60 and 100 µM.^31,32^ Additionally, some gels were pre-treated with 100 or 250 µM SAB (Sigma),^33^ diluted in cell culture medium for 30 min before addition of 30 µM MPP^+^ in the presence of SAB.

### C6 toxicity

Using a 96-well plate, 240 µL of collagen solution (prepared as above) containing 3 × 10^6^ C6 cells/mL collagen was seeded in each well and maintained in culture medium and incubated for 24 h. The medium was removed, and gels were then stabilised using RAFT absorbers (Lonza Bioscience) for 15 min. Following a further 3 days of incubation in the medium, gels were exposed to okadaic acid at concentrations of 5, 25 and 50 nM and MPP^+^ at concentrations of 1, 10, 30, 60 and 100 µM for 24 h, prepared as previously stated. Cell death was assessed using propidium iodide (PI; Sigma) staining in combination with Hoechst 33258 (1 µg/mL; Sigma). PI was added to cultures at 200 µg/mL in cell culture medium and left to incubate for 15 min at 37°C. The medium was then removed, and the cultures were rinsed in Ham’s F12 culture medium and then phosphate-buffered saline (PBS) before fixation. Gels were incubated with Hoechst 33258 in PBS for 10 min, before three times of 5-min washes in PBS, and analysed on a fluorescence microscope (Leica DMIRB). Images were taken from three random areas of each gel, and a cell count was obtained to give a mean percentage of cell death.

### Immunocytochemistry

Following fixation, cell permeabilisation was performed using 0.5% TritonX-100 (Sigma) for 30 min. Following three times of 5-min washes, non-specific binding was blocked with 5% normal goat serum (Dako, Ely, UK) in PBS for 30 min. After another wash step, primary antibodies diluted in PBS (mouse anti-β-tubulin III IgG; 1:400 (Sigma), rabbit anti-glial fibrillary acidic protein (GFAP); 1:300 (Dako)) were applied to gels and incubated overnight at 4°C. Following three times of 10-min washes, secondary antibodies (anti-mouse DyLight 488 and anti-rabbit DyLight 549; 1:300; Vector Laboratories) diluted in PBS were added for 90 min. Hoechst 33258 was also added into the secondary antibody dilutions for cell counting. Omission of primary or secondary antibody was routinely used as a control.

### Image analysis and quantification

Confocal microscopy (Biorad confocal fitted to Olympus BX51 upright microscope) was used to capture images of neurites from five pre-determined areas of each gel using a 20× lens and z stacks of typically 25 µm depth. The length of each neurite was measured using ImageJ.

### Assessment of cellular alignment

Confocal microscopy was used in the assessment of cellular alignment in hydrogels. For EngNT co-cultures created with 1-mL gel, six areas were sampled from the mid and side regions of the gel, as described in O’Rourke et al.,^30^ using a 20× lens and z stacks of 100 µm depth typically. For those created with 50 µL gel, z stacks were set to sample the entire depth, and the gel was sampled using a tile scan method to create a 3D image of the entire gel.

Image analysis was conducted using Volocity™ 6.4 (PerkinElmer) running automated 3D image analysis protocols to measure the angle of cell alignment.^25^ Volocity recognises glial cell processes or neurites based on fluorescence intensity thresholds and approximates them to a straight line using the skeletal length function, allowing the length (µm) and bearing (degrees) relative to the long axis of the gel to be quantified.^34,35^

### Statistical analysis

Normality tests were performed on all data to determine which test was appropriate, and one-way analysis of variance (ANOVA) or t-tests were performed, as data followed a normal distribution. A one-way ANOVA was followed by a Dunnett’s post hoc test to compare multiple conditions against the control. For all tests, *p <0.05, **p <0.01 and ***p <0.001 were considered to be significant.

## Results

### Okadaic acid induces neurite degeneration of PC12 and DRG neurons in a dose-dependent manner

**Figure 1.**
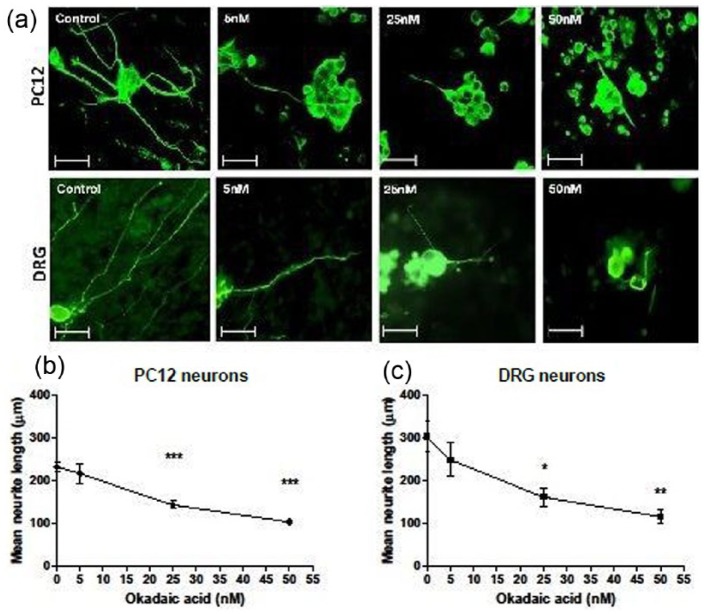
Okadaic acid induces neurite degeneration in PC12 and DRG neurons. (a) Confocal micrographs of 1 mL EngNT cultures show progressive degeneration of PC12 and DRG neurites stained for β-III tubulin (green) after exposure to okadaic acid for 24 h. Scale bar = 100 µm. Dose-dependent neurite retraction was observed in both (b) PC12 and (c) DRG neurons after 24 h of exposure to okadaic acid. Significant differences in neurite length were seen in the presence of 25 and 50 nM okadaic acid when compared to controls. N = 5 gels (PC12), N = 4 gels (DRG), mean ± SEM for each condition. One-way ANOVA with Dunnett’s post hoc test, *p < 0.05, **p < 0.01, ***p < 0.001.

To investigate the effect of okadaic acid on PC12 and DRG neurons, EngNT co-cultures created within a 1 mL mould with both cell types were incubated with various concentrations (5, 25 and 50 nM) of okadaic acid for 24 h. A dose-dependent decrease in neurite length was observed for both cell types. Confocal micrographs depicted in Figure 1(a) illustrate the change in neurite length in response to the presence of okadaic acid. A significant reduction in comparison to the control was observed at concentrations of 25 and 50 nM (Figure 1(b), 1(b)).

### MPP^+^ induces neurite degeneration of PC12 and DRG neurons in a dose-dependent manner

**Figure 2.**
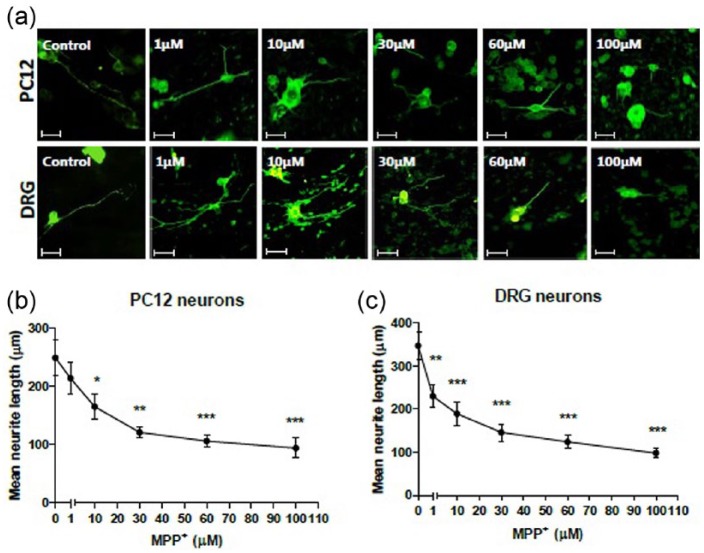
MPP+ induces neurite degeneration in PC12 and DRG neurons. (a) Confocal micrographs of PC12 and DRG neurite degeneration after exposure to MPP^+^ at various concentrations for 24 h within 1 mL EngNT cultures stained to detect β-III tubulin immunoreactivity (green). Scale bar = 100 µm. 24 h of exposure to MPP^+^-induced dose-dependent neurite retraction of aligned (b) PC12 and (c) primary DRG neurons. Gels treated with neurotoxins were compared to untreated control gels. N = 5 gels (PC12), N = 4 gels (DRG), mean ± SEM for each condition. One-way ANOVA with Dunnett’s post hoc test, *p < 0.05, **p < 0.01 ***p < 0.001.

Similar to okadaic acid, the effect of MPP^+^ was investigated following incubation of EngNT co-cultures created within a 1-mL mould using both PC12 and DRG neurons. Following exposure to concentrations of 1, 10, 30, 60 and 100 µM MPP^+^, a dose-dependent decrease in neurite length was observed for both cell types after 24 h. Confocal micrographs depicted in Figure 2(a) illustrate the change in neurite length in response to the presence of MPP^+^. For DRG neurons, all concentrations of MPP^+^ resulted in a significant decrease in neurite length when compared to the control (Figure 2(c)), while for PC12 cells a significant decrease relative to control was observed for all concentrations except for 1 µM (Figure 2(b)).

### C6 glial cell viability is largely unaffected by exposure to neurotoxins

**Figure 3.**
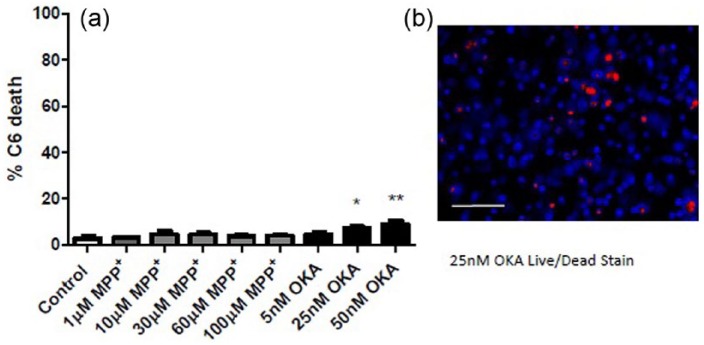
Neurotoxins have a minimal toxic effect on C6 glia. (a) Incubation of gels containing C6 cells with 25 and 50 nM okadaic acid resulted in a significant increase in cell death when compared to the control. However, in all other conditions, the level of cell death did not differ significantly from that in control cultures. Mean ± SEM, n = 5 gels per concentration. One-way ANOVA with Dunnett’s post hoc test, *p < 0.05, **p < 0.01. (b) Fluorescent micrograph show nuclei staining of C6 cells (blue) and PI staining (red) of dead cells in the presence of 25 nM OKA. Scale bar = 100 µm.

Collagen gels containing C6 glia set in a 96-well plate were incubated with either okadaic acid (5, 25 or 50 nM) or MPP^+^ (1, 10, 30, 60 or 100 µM) to investigate the direct effect of these neurotoxins on the glial cells. The presence of MPP^+^ did not appear to increase C6 cell death, with no significant difference seen for all concentrations tested compared to the control. The presence of 25 and 50 nM okadaic acid, however, appeared to increase C6 cell death by 4% and 6%, as seen in Figure 3. Importantly, average C6 cell death remained under 10% across all conditions.

### SAB prevents MPP^+^-induced neurite degeneration in PC12 cells

**Figure 4.**
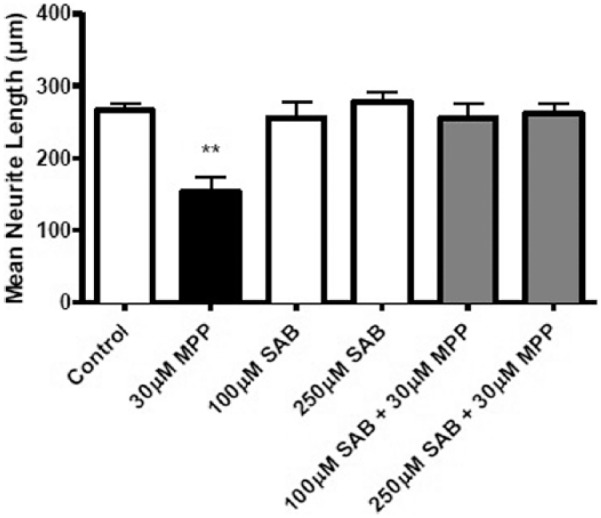
Salvianolic acid B (SAB) prevents MPP^+^-induced neurite degeneration in PC12 cells. Following 30 min pre-treatment of 1 mL EngNT co-cultures and presence of SAB for 24 h, mean neurite length of gels treated with SAB was not significantly different from the control, while MPP^+^ exposure with no SAB was significantly different from the control. Mean ± SEM, n = 5 gels per concentration. One-way ANOVA with Dunnett’s post hoc test, **p < 0.01.

Pre-treatment of 1 mL EngNT cultures for 30 min and continued presence for 24 h of SAB at concentrations of 100 and 250 µM prevented the reduction in mean neurite length in PC12 cells caused by the presence of MPP^+^. The 30µM MPP+ concentration gives a robust and consistent reduction in neurite length and so was chosen as a suitable treatment for investigation of SAB action. A reduction in neurite length of 56% was seen in the presence of 30 µM MPP^+^, but this reduction was not observed in the presence of SAB treatment, suggesting a protective effect of SAB (Figure 4).

### Development of a stable aligned high-throughput model

**Figure 5.**
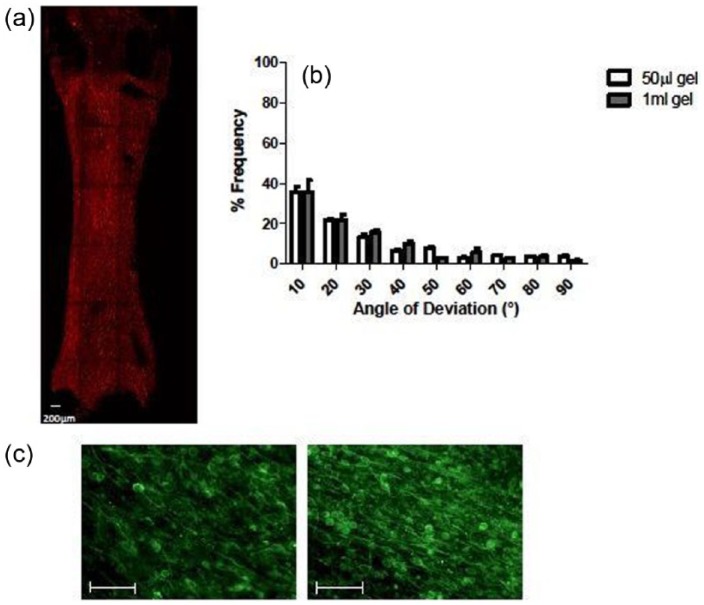
Development of a 50-µL gel for generation of EngNT co-cultures. (a) An example of a tile scan of an entire gel from a 50-µL test rig with seeding density of 3 × 10^6^ C6 cells/mL using confocal microscopy. The C6 cells within the gel were stained for GFAP immunoreactivity. (b) Frequency plot of the 50-µL gel and EngNT in which the angle of deviation was measured to assess alignment, approximately 500 angles per gel were measured. The frequency plots show the % frequency of each angle of deviation in tiles from the mid and side regions of the gel. (c) Confocal projections show the alignment of PC12 cells stained for β-III tubulin immunoreactivity (green) on the surface of scaled down EngNT co-cultures. Scale bar = 100 µm.

Having characterised the behaviour of PC12 and DRG neurons in terms of aligned growth, response to neurotoxins and treatments, the model system was scaled down to fit within the well of a 96-well plate. The resulting aligned gels created within the 50-µL moulds had the dimensions of 6 × 3 mm as seen in Figure 5 (See Supplementary information) and reduced the number of cells and amount of materials required per EngNT by 95% compared to a gel created with the rectangular stainless steel mould. A detailed comparison between the alignment of C6 cells in gels from both types of moulds was undertaken. The angle of deviation (the degree by which C6 processes diverge from the long axis) was measured, and the results revealed that alignment within a 50-µL cellular collagen gel is comparable to that observed within a 1-mL gel (Figure 5). Additionally, PC12 neurons could extend neurites on the surface of the 50-µL gel with a mean neurite length of 200 ± 21 µm, mirroring results seen with a 1-mL gel. This confirms that scaling down EngNT co-cultures does not compromise alignment of glial cells and aligned neurite growth is still supported.

### iPSC-derived neurons extend aligned neurites in EngNT and are sensitive to MPP^+^

**Figure 6.**
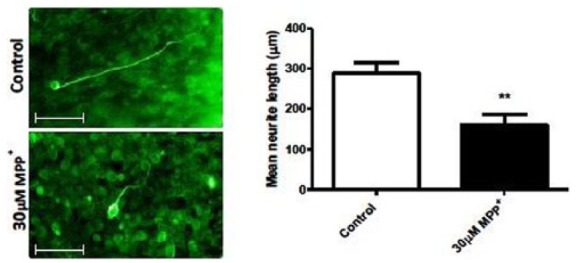
MPP+ induces neurite degeneration of iPSC-derived neurons. Neurite degeneration of aligned iPSC-derived neurons in EngNT co-cultures created using 50-µL gel was observed after 24 h of exposure to 30 µM MPP^+^. Scale bar = 100 µm. Mean ± SEM. Unpaired t-test, **p < 0.01.

The length of neurites extended by iPSC-derived neurons was examined with and without MPP^+^ to determine the applicability of EngNT co-culture created using a 50-µL C6 gel as a tool for the study of neurodegeneration, and whether human iPSC-derived neurons are an appropriate neuronal source (Figure 6). Human iPSC-derived neurons extended neurites on average 300 µm under control conditions, while in the presence of 30 µM MPP^+^ this was significantly reduced to an average of 170 µm, a 40% reduction in neurite length.

## Discussion

There is an unmet requirement for improved in vitro models that more accurately simulate the physical degenerative processes of neurons and allow degeneration to be quantified in a robust and reproducible manner. The aim of this study was to optimise a model system to study neurite retraction in response to neurotoxic compounds, mimicking the early stages of cellular degeneration. The alignment of neurites from PC12, primary DRG and differentiated human iPSC neurons in EngNT co-cultures is supported by the aligned C6 astrocytes within the stabilised collagen gels,^24,25^ and alignment of neurites allows accurate measurement of neurite length and ease of quantification. Neuronal differentiation and neurite formation are important for the development of a neurodegenerative-like pathology, which can be examined in this 3D model system. In vitro 3D structures give a suitable physical and chemical environment which can recapitulate some aspects of this natural niche for neurons and promote physiological neuronal properties.

The results here demonstrated neurite retraction of both PC12 and DRG neurons in a dose-dependent manner as a result of exposure to okadaic acid and MPP^+^. These results are consistent with other studies that have used these compounds to induce neurodegeneration. The effects on the inhibition of neurite growth with exposure to these toxins have been previously established in primary two-dimensional (2D) cell cultures.^36–38^ The neurotoxic effects of okadaic acid have been recognised with observations of degenerating neurons in vitro,^8,9^ and it was demonstrated to induce memory impairment through mitochondrial dysfunction and apoptosis in vivo.^9,10^ MPP^+^ toxicity is widely used in vivo and in vitro for disease modeling.^12,13^ Fang et al. found that treatment with 10 µM MPP^+^ for 24 h caused 39% retraction in primary dopaminergic neurons in vitro, a trend similarly found here using DRGs in EngNT co-culture.^39^

The effect of these neurotoxins on C6 cells were assessed to determine whether neurite retraction was in direct response to the presence of the neurotoxins or in response to glial cell toxicity. The results showed that higher doses of okadaic acid were found to have some toxic effects on glial cells, but cell death did not exceed 10%. This confirms that the neurons were not co-cultured with dying glial cells, indicating that the neurite degeneration observed in the co-cultures was not caused by glial cell death.

SAB (lithospermic acid^40^) is a bioactive component of the red sage plant (*Salvia miltiorrhiza*) that has been researched for its benefits in treating cerebrovascular diseases through antioxidant mechanisms^41^ and also its anti-inflammatory properties.^42^ SAB aids the proliferation of neural stem cells following stroke in rats^43^ and has been found to support myelinated nerves following spinal cord injury^44^ and to promote neuronal cell survival via exerting an anti-inflammatory upon microglia,^45^ indicating a potential neuroprotective effect of this compound. Pre-treatment of EngNT co-cultures for 30 min and subsequent presence of this compound for 24 h significantly prevented the degeneration of neurites exposed to 30 µM MPP^+^. Other studies have also demonstrated the protective effects of SAB against MPP^+^-induced neuronal damage.^46,47^ The use of SAB here further demonstrates its potential neuroprotective effects and illustrates that the model system can be used to test therapeutic compounds.

In this study, robust 3D CNS tissue models engineered by a process of glial cell self-alignment, stabilisation and co-culture with neurons were developed at a scale suitable for high-throughput screening through the development of a 50-µL gel. Characterisation studies that assessed alignment and stabilisation of 50-µL cellular gels created within a 6 × 3 mm mould suggest that a highly organised, stable hydrogel can be created at these dimensions. Interestingly, the dimensions of the 50-µL gel were based on those of a well within a 96-well plate, highlighting that this system could be created in multi-well format.

The potential of EngNT co-cultures created using 50-µL gels for the study of neurodegeneration was confirmed through neurite growth from PC12 cells and iPSC-derived neurons. The results demonstrate that PC12 cells can extend neurites in EngNT co-cultures created using 50-µL gels, as can iPSCs. The response of human iPSC-derived neurons to MPP^+^ was assessed, and these cells demonstrated a significant reduction in neurite length following exposure to MPP^+^ when compared to the control, indicating their suitability for use in studies investigating neurodegeneration. Use of human iPSC-derived neurons in this system creates a physiologically relevant model for studying degenerative conditions. For example, EngNT co-cultures could perhaps incorporate iPSC-derived neurons from patients with neurodegenerative disease, or from specific individuals for stratification or personalised medicine strategies. However, it is important to note that the functional maturation of human iPSC-derived neurons still remains poorly understood, which may limit the usefulness of these cells in modelling adult conditions.

## Conclusion

Recreating key aspects of the 3D environment of the CNS using hydrogel matrices allows neurons and glial cells in vitro to behave similarly to their counterparts in vivo, providing a relevant tool for neurobiological studies. A viable method has been established for the production of engineered tissue, involving cellular self-alignment and subsequent stabilisation in a relatively quick and simple manner to examine neuronal degeneration. The aligned nature of the cells and extracellular matrix in this model system facilitates quantitative analysis of CNS cellular features such as neurite length and degeneration. This simple, consistent and physiologically relevant model system, which uses a multi-well plate format, could potentially be used at a scale suitable for commercial R&D.

## Supplementary Material

Supplementary material
