# Supplementary material for: Adapting tissue-engineered in vitro CNS models for high-throughput study of neurodegeneration
Source: J Tissue Eng. 2017 Mar 15;8:2041731417697920. doi: 10.1177/2041731417697920 (PMC5415290; doi:10.1177/2041731417697920)
Supplement: Supplementary material [file supplementary_data.docx]

**Supplementary Data**

|  | **1ml mould** | **50µl mould** |
| --- | --- | --- |
| *Mould material* | *Stainless steel / PEEK* | *PEEK* |
| *Method of tethering* | *Inserted nylon mesh* | *Integrated tethering bars* |
| *Dimensions of rectangular gel compartment* | *6(w) x 16(l) x 5(h) mm* | *3(w) x 6(l) x 2.5(h) mm* |
| *Volume of gel (including tethering)* | *1ml collagen/cell solution* | *50 µl collagen/cell solution* |

**Table 1 Differences between 1ml mould and 50µl mould for creation of aligned cellular constructs**
